# Whole-Genome Resequencing to Identify Selection Signatures Associated with High Fertility in Lüliang Black Goat

**DOI:** 10.3390/ani15010036

**Published:** 2024-12-26

**Authors:** Xu Wang, Zhenqi Zhou, Xinrui Chai, Jie Li, Wannian Wang, Zhixu Pang, Lifen Cheng, Caihong Cheng, Liying Qiao, Yangyang Pan, Kaijie Yang, Wenzhong Liu, Jianhua Liu

**Affiliations:** 1 Department of Animal Genetics, Breeding and Reproduction, College of Animal Science, Shanxi Agricultural University, Jinzhong 030801, China; wangxupvt@163.com (X.W.); zq204907@163.com (Z.Z.); cc2486143@163.com (X.C.); lijie1124r@163.com (J.L.); wannian1876@163.com (W.W.); pang_z_x@163.com (Z.P.); liyingqiao1970@163.com (L.Q.); panyy@sxau.edu.cn (Y.P.); kjyang@sxau.edu.cn (K.Y.); lwzsxau@gmail.com (W.L.); 2 Shanxi Animal Husbandry Technology Extension Service Center, Taiyuan 030001, China; chlf11@139.com (L.C.); caihong3343@126.com (C.C.); 3 Key Laboratory of Farm Animal Genetic Resources Exploration and Precision Breeding of Shanxi Province, Jinzhong 030801, China

**Keywords:** Lüliang black goat, genetic diversity, signal selection, reproductive traits, kidding numbers

## Abstract

Lüliang black goat (LBG) is a unique livestock genetic resource of great significance for the local agriculture and economic development of Shanxi, China. However, the kidding rate of LBG is relatively low, which limits efforts to improve the reproductive performance and economic benefits of this breed. Therefore, improving the kidding characteristics of LBG is crucial for increasing its economic benefits. In this study, 20 LBG does were selected for whole-genome resequencing and divided into two groups: 10 in the T group (does with three consecutive kiddings of twin kids) and 10 in the S group (does with three consecutive kiddings of single kids). Based on whole-genome resequencing data, this study comprehensively assessed the population structure and genetic diversity of LBG and explored the related genes that affect reproductive performance. Three selection signal analysis methods—Fst (Fixation Index), π (nucleotide diversity), and XP-CLR (Cross Population Composite Likelihood Ratio)—were applied to screen a total of 838 genes, and enrichment analysis was performed to identify genes closely related to the reproductive performance of LBG, including *ENPP3*, *APC*, and *GLI2*. A generalized linear model was used to conduct a correlation analysis between non-synonymous mutations in the three genes and the number of kids produced. Two loci that were significantly correlated with kidding number were identified (*p* < 0.05): *GLI2* g.63400363 C>T and *GLI2* g.63417538 C>T. In general, the LBG population has high genetic diversity and good prospects for genetic improvement. The findings revealed that mining high-fecundity selection characteristics provides a basis for research on goat reproductive mechanisms.

## Simple Summary

Lüliang black goat (LBG) is a unique livestock genetic resource that has strong adaptability and excellent meat quality, and it occupies an important position in agricultural production in Shanxi, China. However, the low litter size and number of LBG has led to a small population. To promote the reproductive performance and economic benefits of LBG, this study used whole-genome resequencing to perform a genetic diversity analysis and found that LBG has high genetic diversity and a certain basis for genetic improvement. We also explored the selection characteristics of LBG in terms of reproductive traits using selection signal analysis. These results provide important genetic information and a theoretical basis for improving LBG reproductive traits, which will aid in the breeding and protection of goats.

## 1. Introduction

Goat (*Capra hircus*) is an important livestock resource, and its kidding trait is not only a key indicator for evaluating reproductive performance but also an important factor that affects the economic benefits of breeding. However, the heritability of kidding number is low (0.08–0.18) [1], making it difficult to achieve significant improvements in the short term through conventional breeding techniques.

With advances in molecular biotechnology, whole-genome resequencing has become a useful method for studying the genetic basis of important economic traits in livestock. These methods allow for accurate sequence determination and abundant genetic variation data collection. In goats, several candidate genes associated with reproductive traits have been identified using whole-genome resequencing. For example, in a study on Shaanbei white cashmere goat, *CDC25C*, *ENDOG*, and *NANOS3* were associated with goat litter size [2]. In a study of Hechuan white goat, Banjiao goat, and Youzhou dark goat, two genes associated with litter size were screened out using whole-genome association analysis: *CSN3* on chromosome 6 and *TCF4* on chromosome 24 [3]. In a study of Australian Boer goat, whole-genome selection signal analysis was used to screen genes related to reproduction, such as *SRD5A1*, *FBXW11*, and *DMRT1* [4]. The discovery of these genes not only indicates the direction for exploring the genetic basis of goat fertility but also provides valuable genetic resources for improving local goat breeds and breeding new breeds.

Mass spectrometry typing is widely used in the livestock industry because it can rapidly and accurately detect candidate gene loci. Examples include the detection and typing of *PRNP* loci in sheep scrapie-related genes [5] and the detection of single nucleotide polymorphisms (SNPs) related to high-temperature resistance in hybrid abalone [6]. In a study on Qianbei Ma goat, mass spectrometry was used to detect four non-synonymous mutations in *CTSS*, and a further association analysis revealed that these sites were significantly associated with the number of kids born (*p* < 0.05) [7]. Therefore, modern molecular biology and genomic technologies, such as whole-genome resequencing and mass spectrometry typing, provide new opportunities and directions for goat multiplicity gene mining and molecular breeding.

Lüliang black goat (LBG) is a unique livestock genetic resource in Shanxi Province, China. It has a long breeding history and produces tender meat. However, the disorderly introduction of species in the last century has led to a sharp decline in the size of the LBG population, even to the brink of extinction. The root cause may be poor kidding performance, with the number of kids (1.16) lower than that in other goats, such as Beetal goat (1.27), Shaanbei white cashmere goat (1.29), and Qianbei Ma goat (2.13) [8,9,10].

Although candidate genes related to various economic traits of goats have been mined, the potential selection characteristics regulating high fertility have not yet been explored, and the biological mechanisms underlying goat kidding are still unclear. Therefore, screening for new candidate regulatory genes related to multiple birth traits remains necessary. For this purpose, we performed whole-genome resequencing of multiple single- and twin-born LBG individuals to identify their high-fertility loci. First, we used principal component analysis to explore their population structure and analyze their genetic diversity. Then, we calculated their genetic differentiation degree through multiple selection signal analysis. Finally, we verified the selected selection characteristics at the population level using mass spectrometry typing technology and association analysis. These results revealed potential selection characteristics related to high fertility in goats, thus providing a new perspective for analyzing the potential genetic mechanisms of goat germplasm characteristics and reproductive performance.

## 2. Materials and Methods

### 2.1. Blood Sample Collection and DNA Extraction

In this study, 221 LBG does with three consecutive kidding records were blooded from the jugular vein groove using 2 mL EDTA negative-pressure anticoagulation tubes, and genomic DNA was isolated using the standard phenol–chloroform extraction method. Each blood sample was lysed and centrifuged (450× *g* for 10 min) at least three times using red blood cell lysis buffer to obtain a white precipitate. DNA was extracted using phenol/chloroform/isoamyl alcohol (25:24:1) reagent. The concentration and purity of the extracted DNA were determined using a NanoDrop 2000 spectrophotometer (Thermo Fisher Scientific, Waltham, Massachusetts, USA) and 1% agarose gel electrophoresis to ensure the quality of the results. The blood samples and DNA samples collected in this study were stored at −80 °C.

### 2.2. Sample Selection and Whole-Genome Resequencing

Among the 221 LBG does with three consecutive kidding records, 10 does that gave birth to twins and 10 does that gave birth to single kids for three consecutive litters were selected and defined as the twin doe group (T) and single doe group (S), respectively. The DNA samples were subjected to whole-genome resequencing at Beijing Novogene Technology Co., Ltd. (Beijing, China) using the Illumina NovaSeq 6000 platform.

### 2.3. Data Preprocessing and Quality Control

We performed strict quality control on the whole-genome resequencing data. Specifically, during the data preprocessing process, fragments containing adapter sequences, fragments with base N content exceeding 10% of the length ratio, and low-quality (quality value Q ≤ 5) fragments with bases exceeding 50% of the length ratio were removed. Subsequently, the results were aligned to the reference genome (https://ftp.ncbi.nlm.nih.gov/genomes/all/GCF/001/704/415/GCF_001704415.2_ARS1.2/GCF_001704415.2_ARS1.2_genomic.fna.gz accessed on 2 September 2022) using BWA v0.7.8 software [11] with the parameters “mem -t 4 -k 32 -M”. The alignment results were removed using SAMTOOLS v1.3.1 [12].

SAMTOOLS v1.3.1 software was used again for population SNP detection, which retained high-quality SNP sites with a minimum sequencing depth of more than 5, a missing ratio of less than 0.1, and a minimum allele frequency of more than 0.05 for a single sample. ANNOVAR v1.3.1 software [13] was used to annotate the SNP detection results.

### 2.4. Genetic Structure and Genetic Diversity Analysis

We performed principal component analysis on the 20 LBG individuals mentioned above using GCTA v1.94.1 [14] to assess their genetic structure. Subsequently, we calculated genetic diversity indices, namely, the observed heterozygosity (*H_O_*), expected heterozygosity (*He*), minor allele frequency (MAF), proportion of polymorphic markers (*P_N_*), polymorphic information content (*PIC*), and effective number of alleles (*Ae*), using PLINK v1.90. In addition, PopLDdecay v3.42 [15,16] was used to determine the linkage disequilibrium (LD) decay. The results were visualized using the R package “ggplot2” v3.5.0.

Runs of homozygosity (ROH) detection was performed on each goat using the R package detectRUNS v0.9.6 [17]. The specific criteria were as follows: set the sliding window to 50 SNPs; allow up to one heterozygous genotype per window; and set the significance threshold to 0.05, the maximum gap between consecutive SNPs to 300 kb, the minimum SNP density to every 50 kb for at least one SNP, and the minimum ROH length to 300 kb. The ROH lengths were divided into five categories: <1 Mb, 1–5 Mb, 5–10 Mb, 10–20 Mb, and >20 Mb.

### 2.5. Selection Signal Analysis

We used three methods to perform selection signal analysis and identify potential selection signatures in the T group. Paired Fst values and −log2(θπ_T/θπ_S) values were calculated genome-wide using the VCFtools v0.1.13 program [18], with a sliding window of 50 kb and a step size of 10 kb. A composite likelihood ratio test (XP-CLR) was performed across populations using the XP-CLR v1.1.2 program [19], with the same sliding window and step size. Subsequently, strong selection regions were screened using a threshold of 5% and mapped to the goat reference genome (ARS1.2) for gene annotation to identify genes associated with multiplicity.

### 2.6. Enrichment Analysis

Genes annotated using the three methods were selected, and Gene Ontology (GO) and Kyoto Encyclopedia of Genes and Genomes (KEGG) functional enrichment analyses were then performed using DAVID (https://david.ncifcrf.gov/ accessed on 12 February 2023) to screen for candidate genes and pathways that regulate litter size.

### 2.7. Mass Spectrometry Typing

Assay Design 3.1 software was used to design primer pairs for non-synonymous mutation sites on candidate genes. Genotypes were tested using DNA samples from 201 does (The testing was completed by Beijing Compson Biotechnology Co., Ltd., Beijing, China), and sites with non-100% detection rates and low numbers of heterozygotes were excluded.

### 2.8. Association Analysis

The following generalized linear model (GLM) [20] was used to analyze the correlation between SNP loci and kid size:

(1) $$Y=\mu +W\alpha +X_{s}\beta_{s}+e,$$

where Y is the number of kids born; μ is the population mean; $X_{s}$ is the vector of genotype values of the s-th SNP; $\beta_{s}$ is its effect; W represents other covariates (parity: 1, 2, and 3); α is their effect; and e is the random residual.

## 3. Results

### 3.1. Quality Control to Obtain Qualified Whole-Genome Resequencing Data

**Figure 1:**
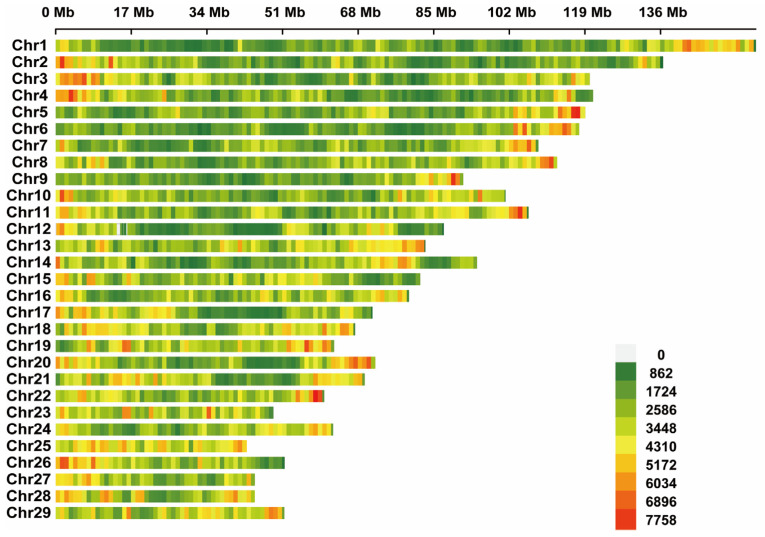
Single nucleotide polymorphism (SNP) density distribution map of the Lüliang black goat (LBG) population.

By performing quality control on the whole-genome resequencing data of 20 samples from the T and S groups, a total of 647.09 Gb of high-quality data was obtained. The sequencing quality was high (Q20 ≥ 90%, Q30 ≥ 85%), GC content distribution was normal, and there was no contamination detected in any of the 20 samples. These results indicate that the library construction and sequencing were successful (Table S1). The average alignment rate of the population samples was 99.19%, with an average genome sequencing depth of 9.51× and an average coverage of 94.98% (Table S2). After high-standard quality control, a total of 7,329,129 SNPs were retained, and the SNP distribution in 29 pairs of goat autosomes is shown in Figure 1. ANNOVAR annotation revealed that the majority of mutations were intergenic and intronic with 4,124,032 and 2,673,507 mutations, respectively. In the exons, UTR′3 was the main mutation, with a total of 54,048 mutations. The transition-to-transversion ratio was calculated to be 2.51 (Table 1).

### 3.2. Genetic Structure and Genetic Diversity of the LBG Population

The genetic diversity of the two groups, T and S groups, was assessed based on six diversity indices. The results are presented in Table 2. The *H_O_* was 0.325 ± 0.212 in the T group and 0.318 ± 0.209 in the S group. The *He* was similar between the two groups, with values of 0.298 ± 0.161 for the T group and 0.296 ± 0.161 for the S group. The MAF was also similar between the groups, with values of 0.223 ± 0.156 for the T group and 0.222 ± 0.156 for the S group. Both groups showed a high *PN*, with values of 0.984 for the T group and 0.983 for the S group. The *PIC* was 0.298 ± 0.161 for the T group and 0.296 ± 0.161 for the S group. The *Ae* was 1.503 ± 0.343 for the T group and 1.500 ± 0.343 for the S group.

**Figure 2:**
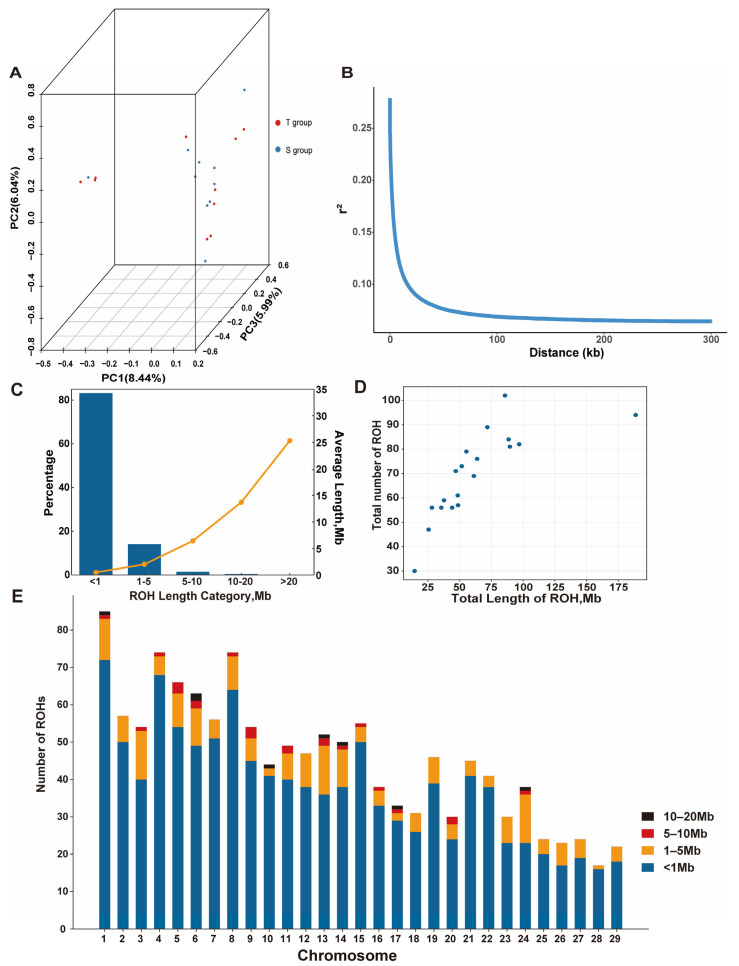
Genetic diversity analysis of the Lüliang black goat (LBG) population. (**A**) PCA of LBG population; (**B**) linkage disequilibrium (LD) decay plot of the LBG population; (**C**) frequency (bar graph) and average length (orange line) of ROHs in different length categories; (**D**) total length of ROHs and total number of ROHs for per goat; (**E**) number of ROHs in the different ROH length categories on each chromosome in the LBG population.

Principal component analysis (PCA) revealed no significant stratification within the LBG population. The genetic distance between the T and S groups was close, indicating a small genetic difference. The first two principal components (PC1 and PC2) explained 8.44% and 6.04% of the total variation (Figure 2A).

When the physical distance between SNP sites increased, the LD coefficient (r^2^) in the LBG population gradually decreased (Figure 2B). When the LD decay distance was 1 kb, the average r^2^ reached the highest value (0.99) and then decreased rapidly, falling below 0.1 when the average LD decay distance was 17 kb.

We identified 1322 ROHs in the LBG population. The ROH lengths ranged from 300.02 Kb to 25.30 Mb, with an average length of 894.34 Kb. Among them, ROHs with a length of <1 Mb accounted for the largest proportion (83.36%). The frequencies of ROHs with lengths of 1–5 Mb, 5–10 Mb, 10–20 Mb, and >20 Mb were 14.29%, 1.66%, 0.61%, and 0.08%, respectively (Figure 2C).

For LBG individuals, the average ROH length ranged from 2.91 to 32.13 Mb. Among them, S group-2 contained 94 ROHs, and the total length of the ROHs (188.70 Mb) was significantly longer than that of the other individuals. T group-9 had the shortest total ROH length (14.46 Mb) and contained only 30 ROHs (Figure 2D). Moreover, chromosome 1 had the largest number of ROHs (85), and chromosome 28 had the least number of ROHs (17) (Figure 2E).

### 3.3. Identification of Selection Characteristics of the T and S Groups by Selection Signal Analysis

**Figure 3:**
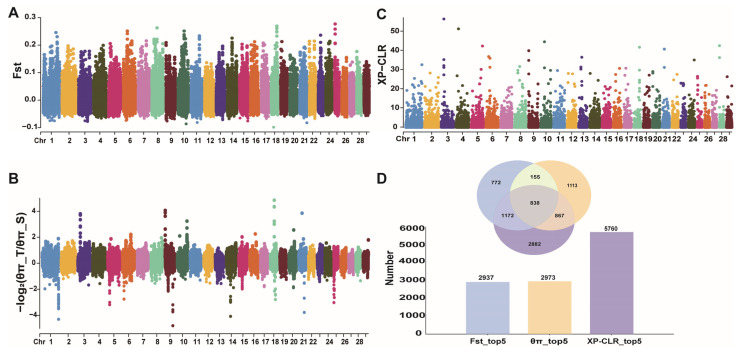
Manhattan plots of the selection signatures detected in the Lüliang black goat (LBG) population by the genetic differentiation index (Fst) (**A**), nucleotide diversity (π) (**B**), and cross-population composite likelihood ratio (XP-CLR) (**C**) methods; (**D**) number of genes annotated by three methods.

We compared the genomes of the T and S groups to identify the genomic regions that affected the kidding number. By scanning the genomic regions with a higher genetic differentiation index (Fst) between the groups, the top 5% of the T group Fst values (0.05) included 10,528 regions (Figure 3A). By calculating the nucleotide diversity (π) in their genomes and selecting windows with the top 5% diversity ratio (0.45), 9266 regions were found (Figure 3B). In addition, genomic regions with extreme allele frequency differentiation were screened using the cross-population composite likelihood ratio (XP-CLR) test. The top 5% XP-CLR value (0.98) identified 9465 regions in the T group (Figure 3C). After annotation and removal of duplicate genes, Fst, π, and XP-CLR were performed and screened 2937, 2973, and 5760 genes, respectively, of which 838 genes were annotated together (Table S4).

### 3.4. Enrichment Analysis of Genes Screened out by T Group

**Figure 4:**
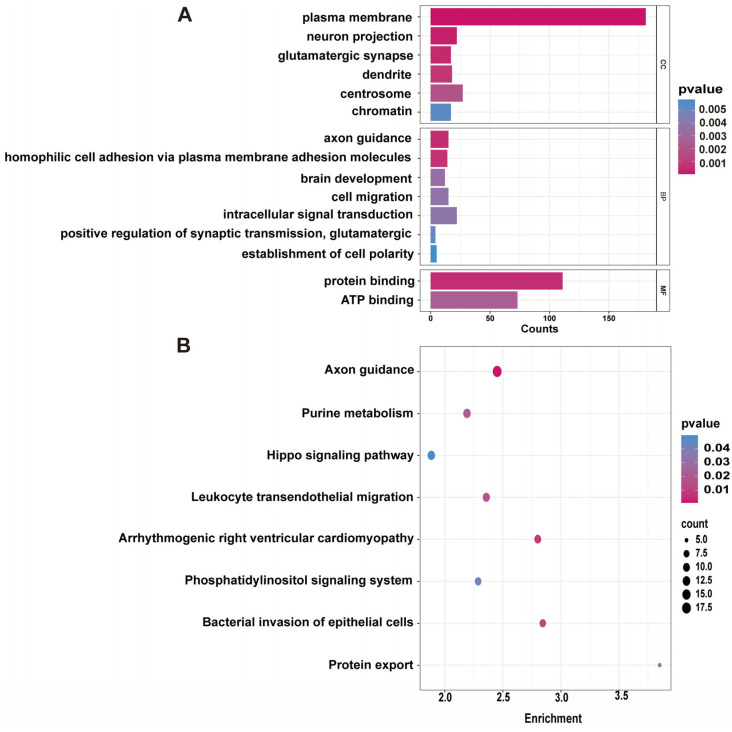
Gene Ontology (GO) and Kyoto Encyclopedia of Genes and Genomes (KEGG) enrichment analysis of the Lüliang black goat (LBG) population. (**A**) GO enrichment analysis results for the first 15 genes. (**B**) KEGG pathway analysis results for the LBG population.

We performed an enrichment analysis on the 838 candidate genes for litter size that were jointly annotated using the three methods. We identified 69 significantly enriched (*p* < 0.05) GO terms (Figure 4A, Table S4), including “axon guidance”, “homophilic cell adhesion via plasma membrane adhesion molecules”, “brain development”, “plasma membrane”, “neuron projection”, “glutamatergic synapse”, “protein binding”, and “ATP binding”. We also found eight significantly enriched (*p* < 0.05) KEGG pathways (Figure 4B, Table S4), including “axon guidance”, “arrhythmogenic right ventricular cardiomyopathy”, and “bacterial invasion of epithelial cells”. In addition, among these 838 genes, *ENPP3* was enriched in the “plasma membrane”, while *APC* and *GLI2* were enriched in the “Hippo signaling pathway”, both of which are closely associated with reproduction. 

### 3.5. Detection and Mass Spectrometry Typing of SNPs in Genes Related to Litter Size

Based on the resequencing data, we identified 17 non-synonymous mutations in the *ENPP3*, *APC*, and *GLI2* genes. Among them, five were observed in *ENPP3*, three in *APC*, and nine in *GLI2*. Therefore, we designed primers corresponding to these SNPs and performed mass spectrometry typing (Table S5).

We detected these 17 non-synonymous mutations in 201 LBG, with an average detection rate of 95.26% (Table 3). Among them, an 100% detection rate for eight SNP loci, and SNP8 was eliminated because it only used two genotypes, and there were only four heterozygotes in 201 individuals. Finally, SNP2, 3, 4, 6, 7, 15, and 16 were retained for subsequent association analysis.

### 3.6. Association Analysis Between SNP Sites and Kidding Number

To evaluate the effects of parity and kidding year on kidding number, we performed analyses to identify significant differences. Regarding the parity effect, the number of kids born in the second and third litters was significantly higher (*p* < 0.05) than the number of kids born in the first litter. For the effect of kidding year, the highest number of kids was recorded in 2021 (1.18 ± 0.042), but there was no significant difference between years (*p* > 0.05) (Table 4). Therefore, we integrated parity effects as covariates into GLMs for subsequent analyses.

After integrating the parity effect into the GLM, an association analysis between the SNP loci and the number of kids was performed. Specifically, SNP15 and SNP16 were extremely significantly associated with the number of kids (*p* < 0.01). Among them, SNP15 had a positive effect with an effect value of 0.20. The number of kids for the TT genotype was the highest (1.44 ± 0.057), which was significantly higher (*p* < 0.01) than that of the CC and CT genotypes (Table 5). In contrast, SNP16 had a negative effect with a value of −0.11, and the number of kids of the TT genotype was the lowest (1.06 ± 0.014), which was significantly lower (*p* < 0.01) than that of the other two genotypes.

The *PIC* of Loci was also identified. In terms of genotype, CC was the dominant genotype for SNP2, SNP6, and SNP8. AA for SNP3, GA for SNP4, GG for SNP7, CT for SNP15, and TT for SNP16 were the dominant genotypes. The *PIC* ranged from 0.02 to 0.35, with an average of 0.24. All seven SNPs conformed to Hardy–Weinberg equilibrium (Table S6).

## 4. Discussion

Population structure and genetic diversity analyses are important for the protection and utilization of LBG. The PCA results showed no obvious stratification of the LBG populations in the T and S groups, indicating that the two groups had similar levels of genetic variation and a common genetic basis. We evaluated the genetic diversity of the LBG population by comprehensively calculating the genetic indicators *H_O_*, *He*, MAF, *P_N_*, *PIC*, *Ae*, LD, and ROH. The genetic diversity analysis reveals that both the T group and S group exhibit similar levels of heterozygosity, allele frequency, and overall genetic variation. Both groups have high proportions of polymorphic markers and similar polymorphism information content, indicating that they are genetically diverse populations. The effective number of alleles is also comparable between the two groups, further supporting the conclusion that their genetic diversity is similar. The results showed that the *H_O_* value (0.322) of the LBG population was slightly higher than the *He* value (0.297), indicating that the heterozygosity level was higher. MAF is an important reference indicator of genetic diversity. SNPs with an MAF higher than 0.3 are generally considered to have high genetic variability [21]. The average MAF value of the LBG population was 0.223, and the proportion of SNPs with an MAF greater than 0.3 was 35.24%, indicating that the LBG population had rich genetic information. The *P_N_* value reached 0.984, further confirming that polymorphisms in the SNP markers in the population were high. The *PIC* was 0.297, indicating that the LBG population had a moderate degree of genetic polymorphisms (0.25 < *PIC* < 0.5) [22]. In addition, the moderate range of *Ae* was between 1.5 and 2.5 [23], and the average *Ae* of the LBG population was 1.502, indicating that the population had relatively rich effective alleles. The LD decay rate and ROH formation rate are usually associated with inbreeding, the magnitude of artificial selection pressure, and the occurrence of genetic drift in the population [24,25,26,27]. In this study, the LBG population showed faster LD decay, and ROHs were mainly short fragments (<1 Mb), indicating that the population had not undergone large-scale inbreeding in recent years and was less subject to artificial selection pressure. In general, the LBG population has high genetic diversity and thus provides a solid genetic basis for population protection and reproductive trait improvements.

We used three selection signal analysis methods (Fst, π, and XP-CLR) to identify potential selection signatures in the T group. Fst revealed broad, large-scale differentiation between the T and S subpopulations, highlighting genomic regions that may have been shaped by divergent selective pressures [28]. π provided a finer view of nucleotide diversity, allowing us to identify regions with reduced diversity that might indicate selective sweeps or bottlenecks [29]. XP-CLR identified more localized selection signatures [30], particularly in regions where allele frequencies differed between the T and S groups, suggesting recent or ongoing selection. Each method provided complementary insights into different aspects of selection within the LBG population. Numerous studies have shown that litter size is affected by follicle maturity and ovulation rate [31,32,33]. In this study, the candidate genes *ENPP3*, *APC*, and *GLI2*, related to reproduction, were screened by the Fst, π, and XP-CLR analysis methods. ENPP3 has not only been identified as a new biomarker for tubal metaplasia but also as a marker gene for endometrial receptivity [34,35]. It also plays an important role in morphological changes and inflammatory responses during ovulation and luteinization, especially during embryo implantation [36]. In addition, *ENPP3* is closely associated with inflammation-related immune responses [37]. *APC* has been widely studied as a cancer suppressor, especially in sporadic colorectal cancer, in which hypermethylation of the CpG island in *APC* is considered one of the main pathogenic factors [38]. Simultaneously, *APC* is involved in regulating the Wnt signaling pathway (Wnt/β-catenin signaling), which plays a role in various developmental processes, such as embryogenesis and regulation of adult tissue homeostasis, regulating cell proliferation, differentiation, stem cell renewal, motility, and apoptosis [39,40,41]. Previous studies indicated that *GLI2* is related to the morphogenesis of hair follicles and the proliferation and apoptosis of dermal papilla cells [42,43] and suggested that *GLI2* mutations may lead to congenital hypopituitarism [44]. In this study, *APC* and *GLI2* were enriched in the Hippo signaling pathway, which not only plays a role in restricting the growth of tissues and organs but also regulates development and tissue/organ homeostasis [45,46]. Other studies have shown that the Hippo pathway plays an important role in early embryonic development [47]. Trophoblasts depend on the low activity of this pathway, whereas inner cell mass formation depends on the high activity of this pathway [48,49]. In summary, the functions of these genes provide preliminary evidence for their potential roles in LBG reproduction.

Identifying genetic markers for high fertility can help select quality breeding stock and improve overall herd productivity. Many studies have explored polymorphisms in *BMPR-1B*, *BMP15*, and *GDF9* in sheep and demonstrated the effects of their loci on ovulation and litter size [50,51,52,53,54]. However, this study only verified 17 non-synonymous mutation SNPs in the LBG population (sample number: 201) and did not study the molecular mechanism. The mass spectrometry results show that 7 SNPs are real and consistent with the Hardy–Weinberg equilibrium state. In the association analysis between kidding number and polymorphisms, two loci (*GLI2* g.63400363 C>T and g.63417538 C>T) showed significant associations (*p* < 0.05). Among these, *GLI2* g.63400363 C>T exhibited a positive correlation with a corresponding effect value of 0.17, while *GLI2* g.63417538 C>T showed a negative correlation with an effect value of −0.12. Given the high genetic diversity within the LBG population, we suggest that future breeding efforts focus on strong artificial selection for the TT genotype of *GLI2* g.63400363 C>T, as well as the CC or CT genotypes of *GLI2* g.63417538 C>T, to improve breeding efficiency and increase kidding rates.

One limitation of our current study is the insufficient production data available for a more comprehensive analysis of fertility traits, such as birth weight, weaning weight, and daily weight gain. Due to the small sample size and the absence of these additional phenotypic measurements, we were unable to perform association analyses for these important traits. While we have focused on the relationship between genetic variations and kidding rates, we recognize that other production traits play a significant role in overall reproductive efficiency and animal performance. We fully intend to address this limitation in future studies by increasing the number of animals included and collecting more detailed phenotypic data. This will allow for a more thorough exploration of the genetic basis of these traits and their potential interactions, which will be crucial for improving both the reproductive and growth performance of the LBG population. We also plan to conduct functional verification of *GLI2* and the two loci by investigating how *GLI2* regulates genes involved in ovarian function, spermatogenesis, and embryonic development. Additionally, we will analyze the differential expression of the *GLI2* gene in various tissues between individuals with the two genotypes and evaluate the expression levels, stability, and functional role of the GLI2 protein in LBGs with different genotypes.

## 5. Conclusions

In this study, whole-genome resequencing was performed to screen candidate genes associated with the number of kids in the LBG, including *ENPP3*, *APC*, and *GLI2*. The high fertility genotypes were TT for *GLI2* g. 63400363 C>T and CC or CT for *GLI2* g. 63417538 C>T. These results provide an important basis for the selection of high-fertility goats and a new direction for improving the reproductive performance of this breed.

## Figures and Tables

**Table 1:** Types and numbers of SNP mutations in the LBG population.

Category	Number of SNPs
Upstream	62,833
Exonic_UTR3	54,048
Exonic_UTR5	15,425
Exonic_UTR5, UTR3	17
Exonic_Stop gain	221
Exonic_Stop loss	42
Exonic_Synonymous	42,400
Exonic_Non-synonymous	26,142
Exonic_unknown	2574
Intronic	2,673,507
Splicing	129
Downstream	68,464
Upstream/downstream	2672
Intergenic	4,124,032
Other	256,623
ts	5,241,890
tv	2,087,239
ts/tv	2.51
Total	7,329,129

SNP: single nucleotide polymorphism; LBG: Lüliang black goat; ts: transition; tv: transversion.

**Table 2:** Six genetic diversity indicators of LBG populations.

Genetic Diversity	T Group (Mean ± SD)	S Group (Mean ± SD)
Heterozygosity Observed (*H_O_*)	0.325 ± 0.212	0.318 ± 0.209
Expected Heterozygosity (*He*)	0.298 ± 0.161	0.296 ± 0.161
Minor Allele Frequency (MAF)	0.223 ± 0.156	0.222 ± 0.156
Proportion of Polymorphic Markers (*PN*)	0.984	0.983
Polymorphism Information Content (*PIC*)	0.298 ± 0.161	0.296 ± 0.161
Effective Numbers of Alleles (*Ae*)	1.503 ± 0.343	1.500 ± 0.343

**Table 3:** Physical locations and call rates of 17 non-synonymous mutations in genes associated with kidding size.

SNP	Genes	Chromosomes	Location	Mutation	Call Rate
SNP1	*ENPP3*	Chr9	56897911	[C/T]	98.91%
SNP2		Chr9	56925452	[C/G]	100.00%
SNP3		Chr9	56943425	[A/C]	100.00%
SNP4		Chr9	56944964	[G/A]	100.00%
SNP5		Chr9	56978677	[A/G]	91.85%
SNP6	*APC*	Chr10	100112427	[G/C]	100.00%
SNP7		Chr10	100113353	[G/A]	100.00%
SNP8		Chr10	100115081	[C/G]	100.00%
SNP9	*GLI2*	Chr2	63381880	[G/A]	98.91%
SNP10		Chr2	63382096	[T/C]	95.65%
SNP11		Chr2	63382358	[G/A]	86.41%
SNP12		Chr2	63382450	[C/T]	82.07%
SNP13		Chr2	63386683	[G/A]	99.46%
SNP14		Chr2	63395465	[T/C]	98.37%
SNP15		Chr2	63400363	[C/T]	100.00%
SNP16		Chr2	63417538	[C/T]	100.00%
SNP17		Chr2	63569359	[T/C]	64.67%

**Table 4:** Analysis of significant differences between parity effect and kidding year effect.

Effect Type		Kidding Numbers (Mean ± SE)	*F* Value	*p* Value
Parity	First	1.11 ± 0.023 ^a^	3.71	0.03
	Second	1.16 ± 0.026 ^ab^		
	Third	1.21 ± 0.029 ^b^		
Years	2018	1.16 ± 0.034	1.73	0.14
	2019	1.14 ± 0.028		
	2020	1.17 ± 0.027		
	2021	1.18 ± 0.042		
	2022	1.14 ± 0.055		

a, b represent different groups. Different letters between groups indicate a significant difference (*p* < 0.05). The same letters between groups indicated no significant differences (*p* > 0.05).

**Table 5:** Site polymorphisms detected at 100%, and their association with litter size.

SNPs	Genotype	Numbers	Kidding Numbers (Mean ± SE)	Genotype Frequency	Effect	*p* Value
SNP2	**CC**	**178**	1.19 ± 0.047	0.89	0.03	0.43
	CG	23	1.16 ± 0.016	0.11		
	GG	0	-	-		
SNP3	**AA**	**165**	1.15 ± 0.016	0.82	0.07	0.69
	CA	32	1.23 ± 0.043	0.16		
	CC	4	1.25 ± 0.131	0.02		
SNP4	GG	35	1.19 ± 0.039	0.17	−0.02	0.55
	**GA**	**89**	1.16 ± 0.023	0.44		
	AA	77	1.15 ± 0.023	0.38		
SNP6	GG	1	1	0.01	−0.03	0.73
	GC	22	1.21 ± 0.050	0.11		
	**CC**	**178**	1.16 ± 0.016	0.89		
SNP7	**GG**	**172**	1.17 ± 0.017	0.86	−0.07	0.37
	GA	28	1.11 ± 0.034	0.14		
	AA	1	1	0.01		
SNP15	**CC**	**80**	1.05 ± 0.015 ^A^	0.40	0.17	0.00
	**CT**	**95**	1.18 ± 0.022 ^B^	0.47		
	TT	26	1.44 ± 0.057 ^C^	0.13		
SNP16	CC	27	1.24 ± 0.047 ^A^	0.13	−0.12	0.00
	CT	84	1.25 ± 0.027 ^A^	0.43		
	**TT**	**90**	1.06 ± 0.014 ^B^	0.45		

A, B, and C represent the different groups. Different letters indicate a highly significant difference between groups (*p* < 0.01). The same letters indicate no significant differences between groups (*p* > 0.05). The bolded parts represent the major genotypes.

## Data Availability

The data presented in this study are available upon request from the corresponding author. The data are not publicly available to preserve privacy.

## Supplementary Materials

The following supporting information can be downloaded at: www.mdpi.com/article/10.3390/ani15010036/s1. Table S1. Quality assessment of 20 LBG sequencing data; Table S2. Sequencing depth and coverage statistics; Table S3. Genes annotated using three selection signals; Table S4. GO and KEGG; Table S5. Site primer design; Table S6. Polymorphisms of the sites for mass spectrometry typing; Figure S1. 17-loci mass spectrometry detection.
